# Diagnostic Accuracy of Oral Fluids Biomarker Profile to Determine the Current and Future Status of Periodontal and Peri-Implant Diseases

**DOI:** 10.3390/diagnostics10100838

**Published:** 2020-10-18

**Authors:** Sarhang S. Gul, Ali A. Abdulkareem, Aram M. Sha, Andrew Rawlinson

**Affiliations:** 1 College of Dentistry, Periodontics Department, University of Sulaimani, Sulaymaniyah 1124–30, Iraq; aram.hamad@univsul.edu.iq; 2 Department of Periodontics, College of Dentistry, University of Baghdad, Baghdad 10011, Iraq; ali.abbas@codental.uobaghdad.edu.iq; 3 Academic Unit of Restorative Dentistry, School of Clinical Dentistry, University of Sheffield, Sheffield S10 2TA, UK; a.rawlinson@sheffield.ac.uk

**Keywords:** periodontal diseases, diagnostic, prognostic, biomarkers, MMP8, point-of-care test

## Abstract

Severe periodontitis is ranked as the sixth most prevalent disease affecting humankind, with an estimated 740 million people affected worldwide. The diagnosis of periodontal diseases mainly relies upon assessment of conventional clinical parameters. However, these parameters reflect past, rather than current, clinical status or future disease progression and, likely, outcome of periodontal treatment. Specific and sensitive biomarkers for periodontal diseases have been examined widely to address these issues and some biomarkers have been translated as point-of-care (PoC) tests. The aim of this review was to provide an update on PoC tests for use in the diagnosis and management of periodontal diseases. Among the PoC tests developed so far, active matrix metalloproteinase-8 has shown promising results in terms of diagnostic and prognostic values. However, further studies are required to increase the sensitivity and specificity via combining more than one biomarker and merging these test kits with periodontal risk assessment tools. Furthermore, the validity of these test kits needs to be investigated by applying the results in further independent studies and the impact on these test kits’, together with the results of risk factors for periodontal diseases, such as diabetes and smoking, also needs to be examined.

## 1. Introduction

Periodontitis is one of the most prevalent chronic inflammatory diseases, characterized clinically by loss of attachment, pathological deepening of the gingival sulcus, and formation of periodontal pockets with resorption of supporting alveolar bone [1]. The initiation and progression of these events are the consequences of an interaction between pathogenic bacteria in the subgingival dental biofilm around teeth and the host response [2]. The effects of periodontitis are not confined locally to the periodontium, and the association with various systemic diseases, such as diabetes, atherosclerosis, cancers, and Alzheimer’s disease, has been suggested [3,4,5,6]. In general, the destruction of periodontal tissues is slow, being characterized by periods of disease activity and remission without obvious alarming symptoms. If neglected, permanent periodontal destruction may occur. Gingivitis may progress to periodontitis and thus, the early diagnosis of gingivitis is an effective way for decreasing the risk of developing periodontitis [7].

Periodontitis is a global public health issue. It affects nearly half of UK adults [8] and is the 6th most predominant disease worldwide, with an overall prevalence of 11.2% affecting around 743 million individuals. The global burden of periodontal disease increased by 57.3% from 1990 to 2010 [9,10,11]. Previous epidemiological studies have found that the highest prevalence of periodontitis is in elderly populations (82%), followed by other adults (73%) and adolescents (59%) [10,11]. People in lower socio-economic groups are disproportionately affected by periodontal disease [9].

Periodontitis is one of the main reasons for tooth loss, which as a consequence can impair mastication, esthetics, self-confidence, and quality of life, as well as socio-economic impacts and increasing healthcare costs [12]. The effect on quality of life is increased by greater extent and severity of periodontal disease [13].

Tissues surrounding dental implants are affected by pathological conditions similar to periodontal diseases affecting the natural dentition [14]. Peri-implant diseases are broadly classified into two clinical conditions, namely peri-implant mucositis and peri-implantitis. Peri-implant mucositis is a reversible inflammatory reaction of the soft tissue around the implant, whereas peri-implantitis is a progressive inflammation extending to the bone surrounding the fixture resulting in bone resorption and, ultimately, loss of the implant [15]. It has been estimated that peri-implantitis affects 7% of dental implants 10 years after placement [16]. Microbiological studies have implicated Gram-negative anaerobic bacteria in the initiation and progression of both peri-implant disease and periodontitis [17].

Internationally, over burdening of the healthcare system economy due to periodontitis has been reported. The global cost of lost productivity suffered from severe periodontitis has been estimated to be 54 billion USD/year [12]. In the UK, the total cost was £2.8 billion in 2008 [18]. The cumulative economic impact of periodontal diseases forms a major component of the 442 billion USD direct and indirect oral diseases costs worldwide [19]. The treatment of peri-implantitis is also costly and, in advanced cases, may necessitate complex treatment using bone substitutes and regenerative techniques [20]. Thus, the treatment of peri-implant disease represents a further potential financial burden to both patients and health care systems.

## 2. Diagnosis of Periodontal Diseases

The diagnosis of periodontal diseases may be defined as the act of recognizing disease from signs and symptoms within the periodontal tissues, indicating a deviation from health. The aim of periodontal disease diagnosis is to determine the type, severity, and location of periodontal destruction. This information gives the basis for effective treatment planning and maintenance care [21]. Currently, the diagnosis of periodontal disease depends largely on the presence and extent of gingival inflammation, which is measured by clinical parameters, including bleeding on probing (BOP) [22], probing pocket depth (PPD) [23], gingival recession, clinical attachment loss (CAL) [24], tooth mobility, and the radiographic pattern and extent of alveolar bone loss. Additionally, consideration is given to age, medical and dental histories, previous treatment, signs and symptoms, including pain, ulceration, and microbial deposits [25]. In clinical practice, the instrument used for periodontal diagnosis is mainly a blunt-ended periodontal probe with millimeter graduations to measure PPD, recession, and CAL, together with BOP. Furthermore, radiographs are used to determine the presence and pattern of alveolar bone loss, which can be detected in people affected by periodontitis.

In 2017, a world consensus from the European Federation of Periodontology (EFP) and the American Academy of Periodontology (AAP) proposed a new classification of periodontal diseases [26]. This sought to add clarity to earlier classifications, taking into account the disease severity/extent and progression by applying a staging and grading system, including both past disease destruction and the intricacy of required therapies [27]. Staging presents the severity of disease, while grading gives additional information about biological characteristics of the disease. For example, a history based assessment of the rate of disease progression, an evaluation of the risk for further progression, the expected response to treatment, and an assessment of the risk that the disease or its therapy may have on oral health are taken into account [27]. However, the new classification system does not focus comprehensively on the definition and criteria for diagnosing peri-implant diseases, which is based mainly on BOP and radiographic evidence of bone loss [26]. These clinical parameters alone are insufficiently reliable to identify active peri-implant disease, future crestal bone loss, or future implant failure [28]. Developing methods for accurate diagnosis and predicting the prognosis of peri-implant disease is of paramount importance for the appropriate clinical management and long-term survival of dental implants.

## 3. Limitations of Traditional Methods for Diagnosis

The traditional methods for diagnosing periodontal diseases are based mainly on the clinical periodontal parameters mentioned above. Supplementary information, such as medical and familial histories, distinctive features, such as bone loss at early age and the amount of dental plaque, is also considered [29]. Whilst this information is helpful, there are a number of limitations. For example, the current diagnostic method is time-consuming and can only indicate past tissue destruction. It does not provide information about the current disease activity nor its future progression and the likely response to treatment.

Furthermore, these clinical methods are subjective, as several factors can affect the outcome of periodontal probing, including the design of the probe tip, the pressure applied by the examiner, the angulation of the probe, inter- or intra-examiner variability, the tolerance of the patient, and whether the examination is performed on a healthy or periodontitis patient [30,31]. A further issue associated with conventional periodontal examination, especially full-mouth probing, is the potential for measurement error due to subjectivity. These drawbacks are further amplified when large-scale epidemiological surveys are conducted that require intra- and inter-examiner calibration. Although the manual periodontal probe is the cheapest and easiest examination instrument, it is not ideal when accuracy and reproducibility are required [30,31]. Additionally, BOP used as an indication of local inflammation, is not a reliable marker for detecting the disease activity, future tissue damage. For example, only approximately 30% of sites that bleed on probing on each of successive examinations are likely to undergo further disease progression [32]. In addition, conventional radiographs only display alveolar bone loss when 30–50% of bone mineral is lost in a particular area [33]. Furthermore, repeat measurement of the above clinical parameters, with the potential for further errors, is performed to monitor clinical outcomes in the management of patients with periodontal and peri-implant diseases [34].

The assessment of dental implants using probing is not without risk, as the force used may jeopardize the vulnerable peri-implant tissues [35,36]. Consequently, radiographic examination of bone loss is also used to assess the status of dental implants. A previous study that utilized an experimental animal model aiming to compare probing forces around natural teeth and dental implants showed that the latter were associated with deeper probe penetration [37]. Furthermore, probing around dental implants cannot be performed until 6 months after loading or placement of the implant, at which time osseointegration is complete [35]. Another limitation is related to the objective of probing around the implant, as it is primarily used to detect the presence of bleeding and/or suppuration [36,37].

Several developments have been introduced to improve the defects of conventional diagnosis, such as pressure-sensitive probes, automated digital probes, and subtraction radiography, that offer more reliable and precise methods for diagnosing periodontal diseases. However, these techniques are mostly used for academic research purposes rather than daily routine clinical practice [38]. Therefore, new diagnostic tests need to be developed which can detect active disease, as well as future disease progression and predict the likely response to periodontal treatment. This information may assist in targeting resources for treatment.

## 4. Need for Alternative Methods with Diagnostic and Prognostic Potential, Such as Use of Biomarkers

Since the 1920s, there have been many changes in the classification of periodontal diseases in an attempt to reach the most accurate diagnosis in order to facilitate treatment planning [26]. The latest classification system aimed to address issues associated with the previous classification system and to provide a standard universal platform that can be easily used by periodontists and general practitioners [39]. Nevertheless, debates and controversies about the current classification system continue, mostly centered around the staging of periodontitis, which may result in confusion about the definitive diagnosis. The intention was to include biomarkers during development of the new classification scheme [39]. However, this was omitted due to the lack of sufficient evidence and global standardized methods for the assessment of biomarkers. The new classification scheme is designed to enable the incorporation of changes in light of future developments [39]. In support for this notion, a recent study aimed to incorporate a point-of-care (PoC) mouth rinse test into the new classification system. The authors have suggested the usefulness of this test as a rapid and effective adjunct diagnostic tool in determining grading and staging of periodontitis [40].

The limitations associated with traditional diagnostic methods, has led to research into laboratory and chairside tests. Diagnostic accuracy, ease of use, and low cost are important attributes for such tests if these are to become widely used. These criteria have been met in a commercial product, the PoC mouth rinse immunotest, which can measure the level of active matrix metalloproteinase 8 (aMMP8) within 5 min and assist in diagnosis and predicting the prognosis of periodontitis [41,42]. Despite the revolutionary concept of using a chair-side PoC test and the advances that have been achieved, the use of these novel assays remains in the research field, and their application in routine dental practice is limited [43].

**Figure 1:**
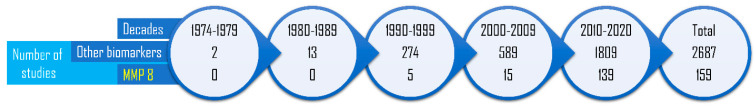
Number of studies examined biomarkers for periodontal diseases from 1974 to 2020 (PubMed).

A search of the literature reveals the number of studies examining biomarkers in oral fluids as diagnostic tools for periodontal disease has markedly increased in the last decade (Figure 1). MMP8 is shown separately here due to its importance. It is anticipated that increased understanding of biomarkers in periodontal health and disease will lead to the further development of chair-side technologies enabling dental practitioners to diagnose periodontal diseases and to predict the prognosis and responsiveness to periodontal therapy. Furthermore, biomarkers may be useful in screening as an adjunct in epidemiological studies.

## 5. Sources of Biomarkers of Periodontal Disease in the Oral Cavity

Saliva, gingival crevicular fluid (GCF), peri-implant sulcular fluid (PISF), and mouth rinse remnant constitute reliable sources of biomarkers in the oral cavity that are readily available. These fluids may be collected non-invasively, with a high potential to reflect periodontal health and disease status through examining the biomarkers within them [44]. However, certain limitations affect the quality and quantity of each fluid collected. Several methods have been described for the collection of GCF, such as absorption onto paper strips, microcapillary pipetting, and sulcular washing methods [45,46]. Despite the fact that GCF provides high levels of different biomarkers, the volume of this fluid is drastically altered in response to health or disease [47]. This fluctuation greatly influences collection time by microcapillary pipetting, which ranges from 10 min for diseased sites up to 40 min in healthy sites [48,49]. In contrast, absorption methods require relatively much less time, for example, 30–60 s for collecting GCF/PISF. However, variation in the manufactured quality of the paper points or paper strips used could affect their absorptive efficiency, and difficulties may also occur in retrieving the target molecules from the paper-strips/points [46,50]. Furthermore, using a washing technique is technically demanding and is usually associated with gingival irritation [50]. In general, with all methods for GCF/PISF collection, there is the possibility of contaminating the sample with blood or saliva that could affect the test outcome and would require repeating the sampling procedure [46].

Saliva is rich in a wide array of biomarkers that can be easily collected and stored in larger amounts than GCF without any potential trauma to the periodontal tissues [51]. Errors associated with interpretation of salivary samples are mostly related to variations in the volume and composition of saliva. These may be due to differences in pathological and physiological states between individuals, as well as within the same person at different times. These make the standardization and quantification of biomarkers difficult when collecting cross-sectional or longitudinal samples [52,53]. In addition, the presence of other elements in saliva, such as mucin and cell debris, can make it hard to manipulate [54].

The issues related to whole salivary samples can be overcome by collecting mouth rinse samples that provide as accurate results as saliva regarding the discrimination between periodontal health and disease [55]. A further potential sampling variable is that the operator can determine the volume collected, which accordingly may vary between patients. The reliability of the latter fluid as a source of biomarkers (aMMP8, in particular) has been documented in many studies, and it could represent the best option for investigating various biomarkers [41].

## 6. Potential Biomarkers of Periodontal Diseases

**Figure 2:**
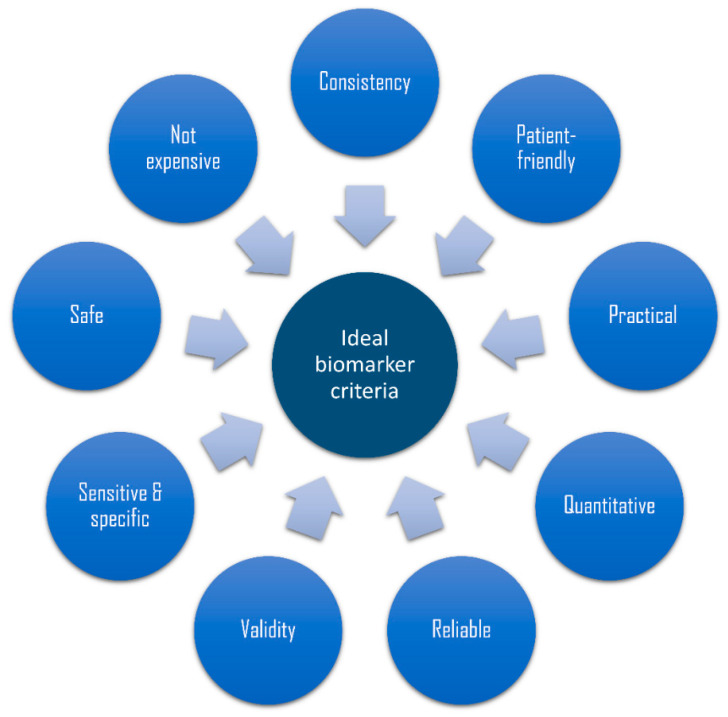
Criteria for the ideal biomarker.

A biomarker was defined by the National Institutes of Health Biomarkers Definitions Working Group as “a characteristic that is objectively measured and evaluated as an indicator of normal biological processes, pathogenic processes, or pharmacologic responses to a therapeutic intervention” [56]. In the last decade, attempts have been made to move periodontal examination from classical methods into using biomarkers that can quantify and qualify relevant clinical information objectively [34]. However, the integrity of certain biomarkers is limited by the availability of specific criteria (Figure 2).

Ideally, the biomarker must be valid, safe to use, easily measured, affordable, and able to be collected non-invasively [56]. In addition, it should be highly sensitive to correctly identify those with disease (true-positive) and specific to precisely identify those without disease (true-negative) [57]. These criteria increase the accuracy of the biomarker as a predictive and diagnostic tool and for efficiently reflecting the patients’ responses to treatment. Furthermore, consistency of results across different ethnic groups, ages, and genders is an important characteristic of an ideal biomarker. This section describes the most promising biomarkers of periodontal and peri-implant diseases.

### 6.1. IL1β

Interleukin (IL)-1β is a potent inflammatory mediator which is critical for the host response to infection or injury. Most of the tissue damage that occurs during chronic or acute diseases or injuries is attributed to this cytokine that is mainly secreted by monocytes and macrophages [58]. Indeed, IL1β is one of the inflammatory mediators highly involved in the pathogenesis of periodontal diseases. Susceptibility of individuals to develop periodontitis was found to be associated with *IL1β gene* (3953/4C>T) polymorphisms [59]. Further, subjects with *IL1β gene* polymorphisms showed increased levels of “orange” and “red” complex periodontal pathogens, which are considered as the main cause of periodontal diseases [60]. It is important to acknowledge that polymorphisms of other *IL1 genes*, such as IL1RN, has been shown to reduce the susceptibility to aggressive and chronic periodontitis via decreasing load of *Porphyromonas gingivalis* (*P. gingivalis*), *Tannerella forsythia* (*T. forsythia*), and *Prevotella intermedia* [61]. Similarly, to the natural dentition, IL1β + 3954C/T genetic polymorphisms were found to be associated with an increased risk of peri-implantitis [62].

The level of IL1β has been found to be significantly and positively associated with increasing PPD and BOP [63]. Analyses of GCF samples from 100 individuals over 12 months have indicated that IL1β is a potential biomarker that can predict periodontal disease progression [64]. A case-control study that monitored salivary IL1β in periodontitis patients before and after phase I treatment showed a significant difference in the level of IL1β between healthy controls and a periodontitis group at baseline [65]. Miller et al. demonstrated that IL1β salivary level significantly increased during periodontal disease compared to controls [66]. Further studies highlighted the ability of IL1β to identify subjects at risk of developing progressive periodontitis and an association of increased IL1β concentration in saliva with increasing rate of bone loss and CAL [66,67].

Utilization of this cytokine as a biochemical marker to distinguish between disease and health status of peri-implant tissue has been demonstrated by several studies. Assessment of IL1β level in PISF over 12 months suggested its usefulness as adjunct to clinical parameters and radiographs in detecting early inflammation around dental implants [68]. Additionally, levels of IL1β were significantly higher in whole salivary samples [69] and PISF samples collected from patients with peri-implantitis, compared with healthy controls [70].

Evidence from available data favors using IL1β as a diagnostic biomarker, predictor of periodontal/peri-implant disease progression, and for monitoring treatment outcomes. However, the use of this biomarker is still limited to the research field, and it has not yet been translated as a chair side test for clinical practice.

### 6.2. IL6 

IL-6 is one of the key acute-phase reactants, and is released by a variety of immune and non-immune cells at the inflammatory site, although macrophages and monocytes are considered as the main source for this cytokine [71]. Involvement of IL-6 in the pathogenesis of periodontal disease is well-recognized. A previous meta-analysis aimed to compare levels of different cytokines in healthy and periodontitis subjects, with response to the treatment indicating increased IL-6 in GCF in the periodontitis group [72]. The susceptibility to periodontitis seems to be increased with *IL-6 gene* 174/G>C polymorphism [73].

Investigation of the IL-6 salivary level in patients with mild, moderate, and severe periodontitis in comparison to healthy controls, showed that IL-6 level proportionally increased with the severity of periodontitis, which suggests the potential diagnostic ability of IL-6 [74]. Another cross-sectional study concluded that IL-6 significantly discriminates between periodontal health and disease in pregnant women [75]. The salivary concentration of IL-6 significantly increased in obese individuals with high cumulative risk score for periodontitis [76]. Furthermore, the level of IL-6 in GCF samples of type 2 diabetes mellitus patients with periodontitis was significantly higher than in systemically-healthy people with or without periodontitis [77]. Comparison of IL-6 level in PISF samples collected from healthy implants and those with peri-implantitis, showed that diseased sites exhibited significantly higher IL-6 levels [70]. Furthermore, levels of IL-6 in whole saliva samples were significantly higher in patients with peri-implantitis than healthy controls [69]. Indeed, IL-6 possesses potential characteristics to be used as a valid, sensitive, and specific biomarker for periodontal/peri-implant disease. However, further studies are required in order to develop a chair-side-PoC test that can be utilized effectively by dentists in clinical settings.

### 6.3. MMP8

Matrix metalloproteinases (MMP) family members are enzymes mainly responsible for degrading all extracellular matrix and basement membrane proteins during physiologic remodeling [78]. During disease, MMP8 is one of the major collagenolytic enzymes highly involved in the destruction of periodontal/peri-implant tissue and progression of periodontitis/peri-implantitis [79]. The level of these biomarkers and MMP8, in particular, were markedly upregulated in proportion to the severity of disease, which potentially makes it possible to measure and accurately reflect the past, current, and anticipated clinical condition [80,81]. Levels of MMP8 considerably increase in oral fluids in association with progressively advancing periodontal/peri-implant diseases [79,80,81]. Recently, the effectiveness of PoC/chair-side testing using saliva, GCF, PISF, and mouth rinse has been comprehensively reviewed [82].

**Figure 3:**
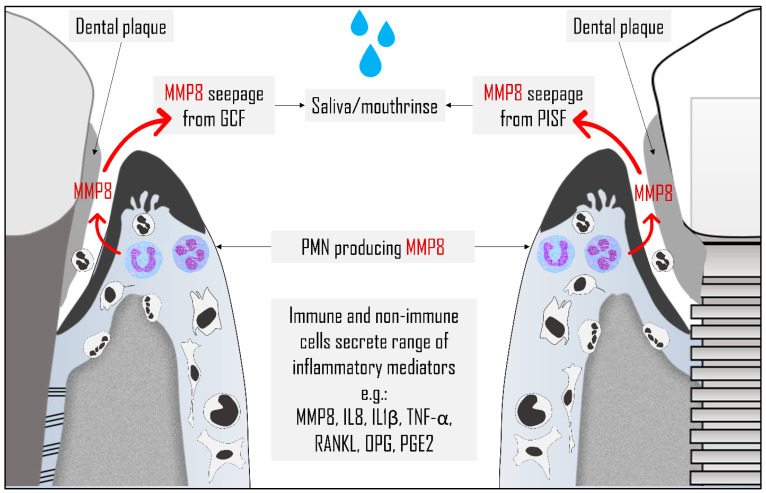
Sources of matrix metalloproteinase (MMP) 8 in oral fluids. Polymorphonuclear leukocytes (PMN) cells represent the main source for MMP8 following their degranulation. MMP8 then released into gingival crevicular fluid (GCF), peri-implant sulcular fluid (PISF), saliva, and mouthrinse samples. MMP8 is also released to a lesser extent from other immune and non-immune cells together with other cytokines, including interleukin (IL) 8, IL1β, tumor necrosis factor (TNF)-α, receptor activator of nuclear factor kappa-B ligand (RANKL), osteoprotegerin (OPG), and prostaglandin E2 (PGE2).

MMP8 is present in oral fluids in detectable amounts that can provide clinically significant and meaningful readings. The main source of this collagenase is degranulated polymorphonuclear leukocytes (PMN) cells, which release up to 20% of their content as MMP8 [83]. On average, each 10^6^ PMN secretes about 60 ng of MMP8, mostly in latent form, which once activated degrades type I and II collagens [79,83,84]. Although PMNs are the major contributors of MMP8 (Figure 3), this collagenase can be derived from other non-PMN lineage sources, including fibroblasts, epithelial cells, endothelial cells, macrophages, and smooth muscle cells [83,85,86]. The salivary MMP8 is derived from PMNs leaking from gingival sulcus to the oral cavity rather than being secreted by major salivary glands [87]. This notion is supported by the high resemblance of salivary MMP8 to GCF/PISF counterparts in its molecular weight (70,000 daltons), with a similar tendency to be activated by gold thioglucose, and lyse the same collagen types [88]. Moreover, the amount of MMP8 was significantly reduced in edentulous subjects when compared to dentate individuals [89].

The presence of pathogenic bacteria, mainly red complex group, in the dental biofilm stimulates production of a range of cytokines, such as tumor necrosis factor-α (TNF-α), IL1β, and MMP8, through Toll-like receptor (TLR) signaling downstream [90]. Among periodontal pathogens, *Treponema denticola* (*T. denticola*) and *T. forsythia* were found to have the potential to induce the inflammatory cascade associated with increased expression of MMP8 [91]. In addition, *T. denticola* and *P. gingivalis* proteases are able to directly activate human pro-collagenases, i.e., by converting latent MMP8 into its activated form [92].

In health, MMP8 in oral fluids is mainly in its latent form, while the expression of the activated form increases in response to periodontal/peri-implant diseases [86,93]. The activity, severity, progression, and response to the treatment of these diseases were found to be significantly and positively associated with the level of aMMP8 [79,94]. Currently, MMP8-based assays are available as chair-side kits that are sensitive, time-saving, specific and accurate in discriminating periodontal health and disease. Indeed, introduction of an MMP8-based point of care test that utilizes saliva as a platform for periodontal disease testing is a “game changer” that not only provides information about the current situation but also identifies susceptible individuals and prognosis of treatment [79,80,95]. A study conducted on Finnish adolescents showed that an active MMP8 PoC test can effectively detect initial periodontitis associated with single nucleotide polymorphisms of *VDR* and *MMP3* genes [96]. Measurement of aMMP8 by lateral-flow chair-side/PoC immunoassay showed that it was highly sensitive to periodontitis, with at least two sites exhibiting PPD ≥5mm [97]. Association of aMMP8 level with clinical parameters [95,97,98] and radiographic findings has been demonstrated by several studies [99,100]. Although a chair-side/PoC aMMP-8 test could not discriminate between smokers and non-smokers with progressive periodontitis [101], it was demonstrated that this assay could effectively predict the prognosis of smokers, in that elevated baseline-MMP8 levels indicated a poor response to treatment [102] and sites that were non-responsive to treatment continued expressing high levels of aMMP8 [101].

Pathologically involved peri-implant sites showed a similar pattern of elevated MMP8 level in PISF to that observed in periodontitis sites [103,104,105], with a similar cellular source being mainly derived from inflammatory cells, particularly PMNs (Figure 3) [105]. Increased severity of bone loss and osteolytic activity during peri-implantitis was found to be associated with the aMMP-8 level in PISF [105], which was further confirmed by another study [106]. Results from a 10 years retrospective analysis, showed a positive correlation between upregulated MMP8 expression in GCF and PISF, and the degree of inflammation [89]. Similar to the natural dentition, the response of tissues supporting dental implants to treatment can be predicted by measuring aMMP-8 levels in oral fluids [85].

**Figure 4:**
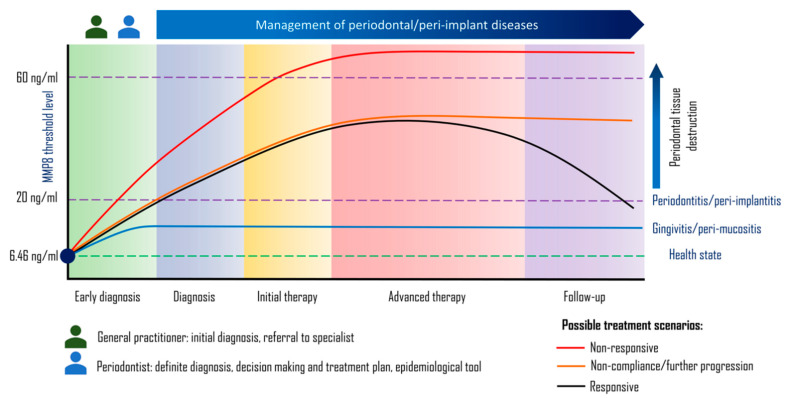
Cut off point of aMMP8 to differentiate periodontal/peri-implant health and disease. The cut off value of aMMP8 that differentiates from health (green dotted line) and gingivitis/peri-mucositis (blue continuous line) is equal to 6.46 ng/mL. While aMMP8 levels ≥20 ng/mL represent the cut off value distinguishing gingivitis/peri-mucositis from periodontitis/peri-implantitis. The latter two conditions could respond favorably to periodontal therapy which is reflected by downregulation of aMMP8 expression in oral fluids (black continuous line) or the destruction of periodontal tissues further progress if neglected (orange continuous line). aMMP8 levels exceeding 60 ng/mL potentially predict poor prognosis of periodontitis/peri-implantitis to periodontal treatment.

Cut-off points of aMMP8 have been determined for a healthy state (<6.46 ng/mL), gingivitis/peri-mucositis (6.64–20 ng/mL), periodontitis/peri-implantitis that respond favorably to the treatment (20–60 ng/mL), and progressive periodontitis that does not respond to the treatment (>60 ng/mL) [107,108,109] (Figure 4). Table 1 summarizes several studies that have investigated the use of MMP8 by different assays for periodontal/peri-implant diseases and which indicate the efficiency of this biomarker in different aspects related to diagnosis and prediction of treatment outcomes for periodontal/peri-implant diseases.

### 6.4. Single vs Combination of Biomarkers

Although some biomarkers can be considered as hallmarks of the current status and progression of periodontal disease [99], certain systemic and local factors may alter the expression of a specific or group of biomarkers, hence compromising their accuracy in diagnosing periodontal diseases. For instance, a recent study has indicated a reduction in the diagnostic accuracy of aMMP-8 PoC oral rinse immunotest in patients affected by Crohn’s disease as compared to systemically-healthy controls [123]. In addition, despite the excellence of MMP8 in differentiating periodontal disease from health state, its level in saliva could be altered due to caries activity [124], increased body mass index (BMI) [125] and smoking [126], which may compromise the diagnostic accuracy, especially in diagnosing early stages of periodontitis. The sensitivity of PoC aMMP8 mouth rinse testing was shown to be lower for single-site pockets and BOP than multiple sites exhibiting bleeding and PPD [97]. Medication may also affect the level of MMP8 expressed, such as host modulation with low doses of certain drugs as an adjunct to conventional treatment(s) of periodontitis/peri-implantitis, mainly aiming to modify destructive aspects of the host inflammatory response [127]. Doxycycline (20 mg) is a well-recognized host response modulator with marked MMP8-inhibitory action [128]. Low dose administration of doxycycline does not cause bacterial resistance, cross-resistance with other antibiotics, or compromise normal flora even after a prolonged intake period, yet, it is effective in counteracting MMP8 [79,128]. Therefore, data from patients using doxycycline should be interpreted with caution, taking into account its inhibitory effect against MMP8.

It has been questioned whether diagnostic accuracy is better when using a single biomarker or biomarker profile. Several studies have been conducted to weigh the advantages of combining different biomarkers over an individual biomarker for predicting and detecting periodontal/peri-implant diseases. The diagnostic accuracy for periodontal disease and its severity was significantly enhanced by measuring multiple salivary biomarkers, including MMP8, MMP9, and osteoprotegerin, together with qPCR, for *P. gingivalis* and *T. denticola* in dental biofilms [129]. Receiver operating characteristic curves analysis has determined the possible thresholds of different biomarkers combined with levels of *P. gingivalis* and *T. forsythia* that possibly distinguish healthy from periodontally-involved sites [119]. A previous study showed that the sensitivity and specificity of aMMP8 alone was greatly diminished in differentiating healthy controls from periodontitis subjects with smoking [126]. However, aMMP8 with other biomarkers, such as tissue inhibitor of matrix metalloproteinases (TIMP) 1 and pyridinoline cross-linked carboxyterminal telopeptide of type I collagen (ICTP), improved the diagnostic accuracy even in smokers [126]. A cohort study that included seven biomarkers showed that IL-1β, IL-6 and MMP-8 combination was the most sensitive and specific for discriminating health from periodontitis [114]. Consistently, synergetic diagnostic accuracy of aMMP8/TIMP1 was also suggested in other studies [112,130]. Gursoy et al. (2011) calculated the cumulative risk score for three salivary biomarkers, IL1β, MMP8, and *P. gingivalis*, for periodontitis patients [131]. The results indicated that these biomarkers can diagnose advanced periodontitis more accurately in combination than on an individual basis [131]. Recently, the cumulative risk score for periodontitis has been strongly associated with microbial biomarker species and salivary humoral immunity [132]. Treatment predictivity of single vs multiple biomarkers was evaluated by previous studies, which concluded that combinations of MMP8, elastase, and sialidase could be more accurate than a single enzyme as prognostic tools [117,119].

## 7. Detection Methods for Biomarkers in Periodontal Diseases

Biomarkers can be used as a basis for the early detection of periodontal disease, future progression, and response to treatment, which can consequently serve for better treatment planning and prognosis. In parallel to the use of different sources (Saliva, GCF, PISF, mouth rinse, and serum) for biomarkers’ measurements, different assays have been used for detection. In general, Immunoassays, such as Immunoblot, immunofluorometric assay (IFMA), ELISA, DentoELISA, and Dento-Analyzer, have been used to determine the levels of biomarkers [100,115]. The presence of high affinity and specific antibodies is the basic communality between these assays [133,134]. Immunoblot is a sensitive method for the detection of biomarkers, especially when discrimination between active and latent forms is required, although determination of the levels of biomarkers is very difficult. Moreover, owing to high cost, the need for specialized equipment, trained staff, and a time-consuming procedure, immunoblot is not adaptable for clinical use [135]. Compared to all assays used, ELISA has been shown to be more sensitive, quantitative and flexible for conducting testing of more than one biomarker in the same sample [136,137]. However, it cannot be used in the dental clinic, as running the assay requires specialized equipment and trained staff. Furthermore, ELISA cannot differentiate the active and latent forms of biomarkers, such as MMP8. This is problematic as the initiation and progression of periodontal disease, and responsiveness to the treatment are more associated with the active form of MMP8 rather than the total enzyme [84,138]. Antibodies that can specifically recognize the active form of MMP8 are of paramount importance.

Substrate degradation assays which measure the active form of an enzyme through degradation of a specific substrate by the enzyme do not require antibodies. The reporter substrate could be a radiolabeled substrate, fluorogenic substrate and change in absorbance in colorimetric assay. Nevertheless, substrate degradation assays also require time and specialized equipment and are, therefore, not adaptable for use in the clinic [139]. On the other hand, detection of periodontal pathogens as biomarkers has been investigated using various test kits as discussed in Section 8. Quantitative PCR is the most sensitive and specific technique used to detect the levels of bacterial pathogens. Translating qPCR into PoC test kits would be of great value in the diagnosis and prognosis of periodontal and peri-implant diseases. However, these are not available currently [140].

In this context, a new PoC technology, “Lab-on-a-Chip” (LOC), has been developed which integrates several laboratory assays in a single miniature device, including sampling procedure, preparation of the sample, detection and measurements of multiple biomarkers, and analysis [141]. The drawbacks of using a combination of biomarkers includes the complexity of interpreting the results and the manufacturing process, together with the considerable cost implications, which contradict the WHO’s ASSURED criteria (affordable, sensitive, specific, user-friendly, rapid and robust, equipment-free and deliverable to end-users) for PoC devices [142]. However, the evidence from the aforementioned studies supports the potential for improvement in diagnostic accuracy by including more than one biomarker in a cost-effective PoC tests.

Recently, two new PoC chairside test kits have been developed, PerioSafe^®^ and ImplantSafe^®^, to detect the level of active MMP8 above 20ng/mL using immune-assay [82]. The kits, similar to pregnancy tests, provide two lines of results indicating higher risk of periodontitis. The advantages of these tests are that they are inexpensive, do not require specialized equipment or trained staff, and provide a quick result within 5 min having high sensitivity and specificity similar to ELISA, which makes them more adaptable for use in clinic. The disadvantage of these PoC test kits is that only a single biomarker is integrated, which is difficult to reflect the complex nature of periodontal diseases [94,108,143,144]. Table 2 summarizes some studies that used PoC aMMP8-chair-side tests to examine periodontal/peri-implant health and disease in oral fluids, as well as that produced promising results in clinical practice.

## 8. Periodontal Point-of-Care Test Kits

PoC technology aims to evaluate the levels of biomarkers that have shown to be associated with the disease status. These tests have already been used in general medicine for blood coagulation, immunological, and cardiovascular biomarkers. Moreover, some of these tests, such as pregnancy tests and for blood glucose levels, are available for home use [150]. There is potential for developing further PoC tests in medicine, and the WHO has introduced the ASSURED criteria for the characteristics of PoC devices. This stipulates that such devices should be “affordable, sensitive, specific, user friendly, rapid, and robust, with no complex equipment and deliverable to end-users” [142].

The development of a PoC test for periodontal diseases that meets the above criteria would be of great value and make life easier for researchers, clinicians, and patients. Since the 1990s, many test kits that have been introduced as prototypes or for commercial use have relied upon chemical, immunological, and microbiological techniques for the evaluation of biomarkers. The idea was to develop a test kit with enhanced diagnostic and prognostic capabilities [151]. This section will review the applicability and usefulness of these kits through the studies that have examined them. In general, the chairside kits can be classified into three groups.

### 8.1. Microbiological Test Kits 

Microbiological test kits have been used to detect periodontopathogenic bacteria that play a role in periodontal diseases, such as *A. actinomycetemcomitans*, *P. gingivalis*, *P. intermedia, T. forsythia*, and *T. denticola.* Evaluation of these bacteria can be used to determine the most common forms of the disease, such as gingivitis and periodontitis (formerly called chronic and aggressive periodontitis). These tests were used to assess the reduction or eradication of periodontal pathogens during periodontal therapy [140]; however, they could not fully satisfy clinical needs. For example, Omnigen diagnostics takes hours to days to perform, Evalusite has very low sensitivity, and PerioScan can only determine the severity of the disease (Table 3).

### 8.2. Biochemical Test Kits

These kits have mainly been used to determine levels of biomarkers in oral fluids. Molecules, such as enzymes (bacterial and host enzymes), mediators of inflammation, and extracellular matrix components that represent the alteration of periodontal tissues have been investigated [38]. Amongst the molecules, enzymes (MMP8 in particular) have been mainly examined and translated as chair side tests. Generally, these tests are not widely used in the clinic because of complex procedure, low sensitivity and specificity [38], whereas, the more recently developed PoC test kits, namely PerioSafe^®^ and ImplantSafe^®^, can provide results within 5–7 min, with sensitivity and specificity of 76.5% and 96.7%, respectively [41,94] (Table 3). 

### 8.3. Genetic Test Kits 

Genetic polymorphisms of IL-1α and IL-1β are likely to be related to an individual’s genetic susceptibility to periodontitis. Although these genes do not cause or initiate the disease, they might enhance earlier development and severity of the periodontitis [140]. GenoTypePST^®^ and MyperioID tests are used to determine the genetic susceptibility to periodontitis (Table 3). 

The biomarkers that have been examined in relation to PoC test kits (Table 3) have been shown to identify the severity of periodontal diseases [54,140,152]. However, apart from PerioSafe^®^ and ImplantSafe^®^, none of these tests have demonstrated the prognostic capabilities of importance to both clinician and patient. Additionally, these tests have been shown not to comply with ASSURED criteria for diagnostic devices [153]. Thus, some of these tests, indeed the majority, are no longer available or rarely used in clinics.

## 9. Clinical Implications and Challenges

In modern dental clinics, the diagnosis of periodontal diseases entirely depends on assessment of clinical parameters of BOP, PPD, CAL, and bone loss. However, these clinical parameters have limitations, including the detection of past rather than current disease activity, lack prognostic value to predict further disease progression, and response to periodontal therapy [153]. Additionally, full mouth clinical assessment is always challenging for clinicians in the dental clinic and for researchers, as it takes time, and, for that reason, basic periodontal examination coding and partial mouth recoding have been developed for clinical and researcher examination, respectively [154,155].

Amongst the PoC test kits, PerioSafe^®^ and ImplantSafe^®^ have shown to be the most reliable and applicable [40,94,108,156]. Their reliability as diagnostic kits are demonstrated by their ability to differentiate gingivitis and periodontitis from healthy periodontium by the cut off value of 6.46 ng/mL. Furthermore, they can differentiate gingivitis and periodontitis by the cut-off point of 20 ng/mL (Figure 4). This is of great value, as these results can be used as a screening tool for patients in the clinic and in research settings where time is limited. In dental clinics, this helps the clinician to exclude the patients that do not require periodontal examination because an MMP8 level <6.46 ng/mL shows that they have a healthy periodontium, or it might encourage the clinician to provide a thorough treatment plan for patients with periodontitis through integrating these test results into the 2017 classification system when the MMP8 level is in the active range from 20–60 ng/mL [40,94,108,156]. In periodontal studies and epidemiological surveys, the prevalence of the most serious form of the disease (periodontitis) could be determined more quickly and accurately by conducting the PerioSafe^®^ test instead of a very time-consuming full mouth periodontal examination of a large number of participants, using a partial mouth protocol or examining representative teeth (CPITN indexed teeth), which have been shown to produce inaccurate results [157]. Even more important is the prognostic value of the PerioSafe^®^ test kit in enabling the clinician to identify patients that are at high risk of further periodontal tissue destruction (MMP8 ranges from 20–60 ng/mL) or not responding to standard periodontal treatment (scaling and root surface debridement) when the active MMP8 level >60 ng/mL [79,94,100,108,146].

General dental practitioners could integrate this test’s results in their referral to a specialist periodontist, which can save time in the referral process and thereby protecting the patients’ periodontal health. For the periodontist, the clinical implications include helping to prioritize treatment and in organizing follow-up appointments. In addition, these tests have the potential to reduce over or under treatment by enabling customized tailored treatment strategies to be developed. For instance, a test result suggesting a patient might not respond to standard periodontal treatment (MMP8 >60 ng/mL), may help the periodontist to decide how best to manage residual sites after initial treatment. This may include the prescription of an adjunctive treatment (local or systemic antibiotic) to accompany further non-surgical treatment or the provision of surgical treatment (open root surface debridement) [42,85,94,143].

Despite the above significant clinical implications, studies that have investigated biomarkers of periodontal diseases have some limitations. For example, the developed test kits are not yet widely accepted by clinicians in routine daily practice [140], which might be related to the fact that clinicians want a diagnostic test that will make a difference in their daily clinical routine, and that is not the case with most of the test kits developed so far, whereas, among the PoC test kits listed above (Table 3), PerioSafe^®^ and ImplantSafe^®^ are compliant with ASSURED criteria and have shown promising results. However, these test kits have shown to be inaccurate in patients with mixed dentition (younger than 15 years old), systemic diseases (Crohn’s diseases) [123], active orthodontic treatment, and mouth ulcers [82]. Finally, variations in sampling technique, assays used, statistical analysis, and data reporting, as well as periodontal diseases case definition, make comparisons between the studies very challenging.

## 10. Conclusions and Future Direction

This review summarized the limitations of traditional clinical parameters, potent periodontal disease biomarkers, and developed PoC test kits used in the diagnosis of periodontal and peri-implant diseases. Biomarker profiles offer the opportunity to obtain a quick overview on present periodontal disease status, future disease progression, and, likely, response to periodontal therapy. The PerioSafe^®^ and ImplantSafe^®^ test kits can be at least a helpful adjunct tool in enhancing the diagnosis and prognosis of periodontal diseases. Future development of PoC test kits should take into account the ASSURED criteria introduced by WHO. Further studies are necessary to increase the diagnostic and prognostic value through combining more than a single biomarker and integrating these test kits into periodontal risk assessment. Furthermore, studies need to be conducted in patients with potential confounders of periodontal disease (such as diabetes mellitus and smoking) and, most importantly, to validate the results with other studies.

## Figures and Tables

**Table 1:** Summary of studies that investigated MMP8 using different assay techniques for periodontal/peri-implant health and disease in oral fluids.

Authors	Aim(s)	Study Groups	Oral Fluids Examined *	Biomarkers, Assays ^†^	Clinical Criteria ^¶^	Results
Ma et al., 2000 [105]	Investigate the correlation between GI, MMP8, and MMP13 level in PISF and amount of peri-implant bone loss	13 patients having implants with different levels of bone resorption	PISF	MMP8 by time-resolved immunofluorometric assay and MMP13 by quantitative immunoblot	Peri-implant vertical bone loss was divided into: <1 mm, 1–3 mm, and >3mm Gingival inflammation recorded on scale from 0–3	Both biomarkers potentially reflect osteolytic process but not GI
Yamazaki-Kubota et al., 2010 [110]	Investigate the level of MMP2, MMP8, and subgingival bacteria in children with Down’s syndrome	Children with DS (*n* = 15) Healthy controls (*n* = 14)	GCF	MMP2 and MMP8 (ELISA)	OHI, PPD, and BOP	Both collagenolytic enzymes were significantly higher in GCF of children with DS than controls even with low oral hygiene index score and absence of BOP.
Rai et al., 2010 [111]	Investigate the level of MMP8 and MMP9 in healthy periodontium and periodontitis	Periodontitis patients (*n* = 10) Healthy controls (*n* = 10)	GCF	Both MMPs measured by ELISA	At least 7 teeth with PPD >6 mm Having at least 12 posterior teeth	Significantly higher level of MMP8 and MMP9 in periodontitis vs healthy controls
Leppilahti et al., 2011 [112]	Assessing the ability of PoC MMP8- mouth rinse, measured by three assays, TIMP1, and elastase activity to distinguish subjects with different periodontal conditions. Comparing assaying methods for MMP8	Randomly selected patients with periodontitis (*n* = 214)	Mouth rinse samples	MMP8 assayed by IFMA, DentoELISA and commercial ELISA. TIMP1 and elastase measured by ELISA	BOP, PPD ≥4 mm, and PIBI	MMP-8/TIMP-1 combination showed higher diagnostic accuracy DentoELISA showed higher sensitivity and specificity in detecting MMP8
Kraft-Neumärker et al., 2012 [113]	Full-mouth analysis to investigate the correlation between clinical parameters and level of MMP8	Females with periodontitis (*n* = 9)	GCF	MMP8 assayed by ELISA	GI, PI, BOP, and PPD (>30% of sites affected)	Increased PPD was associated with increased level of MMP8
Ebersole et al., 2013 [114]	Investigating the level of selected biomarkers in periodontal health and periodontal disease	Healthy (*n* = 30) Periodontitis (*n* = 50)	Saliva	IL1β, IL6, TNFα, and IFNα assayed by human Luminex^®^ multiplex assays MMP8, PGE2, and albumin assayed by ELISA	Healthy: BOP <10% of sites, PPD ≤6 mm in <2% of sites, CAL >2 mm in <1% of sites Periodontitis: PPD ≥5 mm, CAL ≥3 mm, BOP ≥2 in at least 5 sites	Salivary MMP8, IL1β, and IL6 showed the highest diagnostic potential
Leppilahti et al., 2014 [115]	Assess the accuracy of different biomarkers in diagnosing periodontitis Comparing two methods for assaying MMP8	Healthy (*n* = 20 sites) Gingivitis (*n* = 19 sites) Periodontitis (*n* = 19 sites)	GCF	MPO, TIMP1 MMP13, and MMP14. Assayed by ELISA MMP8 Assayed by ELISA and IFMA	Healthy: PPD <3 mm, no CAL, no inflammation Gingivitis: BOP with no loss of attachment Periodontitis: PPD ≥5 mm, CAL ≥3 mm, >50% bone loss in radiograph	MPO and MMP8 can highly discriminate periodontitis. IFMA is more accurate than ELISA for assaying MMP8
Liu and Hwang, 2016 [116]	Assessment of the effect of smoking cessation on periodontal tissue over 12 months	Male smokers joined smoking cessation clinic (*n* = 122)	GCF, Saliva	MMP8, MMP9, and IL1β measured by ELISA, nicotine and cotinine assayed by chromatography-tandem mass spectrometry	PI, GI Patients exhibiting sites >5.5 mm were excluded	The level of MMP8 did not change significantly within the monitoring period between smokers, quit-smokers, oscillators and nonsmokers
Ramseier et al., 2016 [89]	Assessment of biomarkers in PISF 10 years after implant placement	Implants (*n* = 504), adjacent teeth (*n* = 493)	GCF, PISF	MMP8, IL1β, MMP3, MMP1, and MMP1/TIMP1 measured by ELISA	PI, mGI, BOP, PPD, and CAL	Increased level of MMP8 was detected in 90% of sites with progressive inflammation around tooth/implant
Gul et al., 2016 [117]	To assess combined biomarkers compared with single biomarker for predicting the outcome of treatment	Periodontitis patients (*n* = 30)	GCF	Active MMP8, elastase, cathepsin G, trypsin like enzyme and sialidase measured by colorimetric assay	Full mouth PI, BOP, PPD and CAL	Combined active enzyme profiling could provide significant prediction of outcome of treatment.
Kumar et al., 2017 [118]	Evaluate the response of peri-implant connective tissue to titanium and zirconia abutments	Candidates for implant placement (*n* = 12)	PISF	MMP8 assayed by ELISA	GI, PI, and PPD measured by thermoplastic periodontal sensor probe	Titanium abutment induced higher expression of MMP8 at early stages than zirconia
Gul et al., 2017 [119]	Assess the ability of a novel combination of biomarkers to predict treatment outcome of patients with chronic periodontitis	Periodontitis patients (*n* = 77)	GCF and plaque	Active MMP8, elastase and sialidase measured by colorimetric assay, *Pg*, *Tf* and *Td* level determined by qPCR.	Full mouth PI, BOP, PPD and CAL	The ‘‘fingerprint’’ of GCF enzymes and bacteria offers a way to predict the outcome of non-surgical periodontal treatment on a site-specific basis.
Mauramo et al., 2018 [98]	Assessment of association between MMP8 level in oral fluids and periodontitis	Periodontitis patients (*n* = 258)	GCF, Saliva	MMP8 assayed by IFMA	DMFT, BOP, PPD, CAL	MMP8 in saliva and GCF was significantly associated with severity of periodontitis and PPD
Borges et al., 2019 [120]	Investigate attachment loss at sites with progressive periodontal disease following SRP	Periodontitis stage II grade B (*n* = 18) Healthy controls (*n* = 9)	Saliva, GCF	IL-10, MMP8, VEGF, RANKL, OPG and TGF-β1 by Multiplex Cytokine Profiling Assay MMP8 level measured by ELISA	PPD, BOP, and relative CAL	Attachment loss continued in some sites even after SRP. Action of MMP8 seems to be modulated by IL-10
Hong et al., 2020 [107]	Comparing efficiency and accuracy of different biomarkers in diagnosing gingivitis	Healthy (*n* = 15) Gingivitis (*n* = 85)	Saliva and GCF	MMP8, MMP9, cystatin C, MPO, PAF, cathepsin B, lactoferrin, and ICTP ELISA	Gingivitis defined by BOP ≥10%	MMP8 and MPO were significantly and positively correlated with BOP. MMP8 was the most effective in diagnosis of gingivitis
Karteva and Manchorova-Veleva, 2020 [121]	Assessing the accuracy of active (a)MMP8 in diagnosis of asymptomatic apical periodontitis (AAP)	AAP (*n* = 31) Control (*n* = 31)	GCF	aMMP8 by ELISA	CBCT used to confirm the presence of the lesion	Statistically significant increase in aMMP8 level collected from teeth with AAP compared to healthy controls
Liu et al., 2020 [122]	Application of the combined use of baseline salivary biomarkers and clinical parameters in predicting the outcome of scaling and root planning	Advance periodontitis (*n* = 40)	Saliva	MMP8 and IL1β by ELISA, *Pg*, *Aa*, *Pi* and *Tf* by PCR.	PPD, BOP, and CAL	The combination of baseline salivary biomarkers and clinical parameters better predicted SRP outcomes than each alone

* GCF, gingival crevicular fluid; PISF, peri-implant sulcular fluid; ^†^ MMP, matrix metalloproteinase; MPO, myeloperoxidase; PAF, platelet-activating factor; ICTP, pyridinoline cross-linked carboxyterminal telopeptide of type I collagen; IFMA, immunofluorometric assay; IFNα, interferon-α; PGE2, prostaglandin E2; *Pg*, *Porphyromonas gingivalis*; *Aa*, *Aggregatibacter actinomycetemcomitans*; *Pi*, *Prevotella intermedia*; *Tf*, *annerella forsythia*; *Td*, *Treponema denticola; TIMP*, *tissue inhibitors of metalloproteinase*; *IL*, *interleukin*; *TNF*, *tumor necrosis factor*; *VEGF*, *vascular endothelial growth factor*; *RANKL*, *receptor activator of nuclear factor kappa-B ligand*; *OPG*, *osteoprotegerin*; *TGF-B1*, *transforming growth factor beta 1*; ^¶^ PPD, probing pocket depth; CAL, clinical attachment loss; BOP, bleeding on probing; PI, plaque index; GI, gingival index; CBCT, cone beam computed tomography; DMFS, decayed-missed-filled surface index; PBI, papilla bleeding index; OHI, oral hygiene index; mGI, gingival index modified for oral implants; DMFT, decayed, missing and filled teeth; SRP, scaling and root planning.

**Table 2:** Summary of studies that used point-of-care (PoC) aMMP8-chair-side tests to examine oral fluids for periodontal/peri-implant health and disease.

Author, Year	Aim(s)	Study Groups	Oral Fluid Examined	PoC/Chairside Test	Clinical Criteria ^¶^	Results
Mäntylä et al., 2006 [101]	Evaluate the efficacy of MMP8- specific chair-side dip-stick test in longitudinal monitoring of periodontal status of smoker and non-smoker periodontitis patients	Periodontitis patients (*n* = 16)	GCF	MMP8 assayed by chair-side dipstick test	Have at least 20 teeth, BOP, PPD ≥4 mm at 5 or more sites, PI, and CAL	Persistent elevation of MMP8 in GCF may indicate sites at risk and poor response to conventional nonsurgical periodontal treatment
Sorsa et al., 2010 [81]	Comparing four methods to detect MMP8 in GCF	Periodontally healthy (*n* = 2), gingivitis (*n* = 2), moderate-severe periodontitis (*n* = 6)	GCF	IFMA, MMP-8 specific chair-side dip-stick test, Dento-Analyzer, and Amersham ELISA kit	PPD, AL	IFMA and Dento-Analyzer yielded comparable results, followed by chair-side dip-stick test, while Amersham ELISA results were not in line with other assays
Nwhator et al., 2014 [97]	Investigate the clinical correlates of aMMP8-immunotest with BOP, oral hygiene, and PPD	Periodontitis and healthy (*n* = 86), Final analysis included 76 patients	Mouth rinse samples	aMMP8 measured by Lateral flow mouth rinse test (PerioMarker^®^)	BOP, debris index and calculus index scores, and BPE, PPD was charted when BPE score> 3	aMMP8 showed high sensitivity for at least two sites with BOP and two sites with periodontal pockets
Izadi Borujeni et al., 2015 [95]	To evaluate the sensitivity and specificity of aMMP8 PoC immunotest in detecting periodontitis	Untreated generalized periodontitis (*n*=30 equally distributed between moderate and severe cases) Healthy controls (*n* = 30)	Mouth rinse samples	aMMP8 measured by Lateral flow mouth rinse test (PerioMarker^®^)	Moderate periodontitis: PPD ≥3.5mm, CAL =3–4 mm at >30% of sites or ≥5 mm at <30% of sites Severe periodontitis: PPD ≥3.5 mm, CAL ≥5 mm at >30% of sites Healthy: PPD ≤3 mm, CAL ≤2 mm at <30% of sites	aMMP8 positively correlated with generalized periodontitis with diagnostic sensitivity = 87% and specificity = 60%
Heikkinen et al., 2016 [41]	Investigate the ability of PoC aMMP8-mouthrinse to identify adolescents with oral inflammatory burden	Adolescent subjects (*n* = 47)	Mouth rinse samples	aMMP8 measured by Lateral flow mouth rinse test	Full-mouth clinical parameters, including BOP, PI, PPD ≥4mm, and caries sites	aMMP-8 chairside test effectively differentiated adolescents with early initial signs of periodontitis. However, caries was less efficiently detected
Ritzer et al., 2017 [106]	Determine the efficiency of sensory chewing gums as 24/7 detector to differentiate between patients with peri-implant disease and healthy subjects	Peri-implantitis or mucositis group and healthy volunteers	PISF, Unstimulated saliva	aMMP-8 levels were assayed by DentoELISA, Dento-Analyzer, and peptide sensor (PCL ID #1c)	N/A	Level of MMP8 was significantly higher in patients with peri-implant diseases as compared to healthy controls
Heikkinen et al., 2017 [96]	Determination of genetic background for initial periodontitis and caries by PoC aMMP8 immunotest	Adolescent subjects (*n* = 47)	Oral fluid samples	aMMP8 measured by Lateral flow mouth rinse test (PerioSafe^®^)	BOP ≥20% of sites, PPD, PI, and caries status	Genetic polymorphisms of *MMP3* and *VDR* significantly associated with aMMP8 level
Räisänen et al., 2018 [109]	Investigate the effectiveness of aMMP8 PoC immunotest in determining cost-effective treatment(s)	Adolescents (*n* = 47) Adults (*n* = 70)	Mouth rinse samples	aMMP8 measured by Lateral flow mouth rinse test	Treatment need defined by CPITN scores	Results from aMMP8 PoC immunotest were consistent with CPITN codes for treatment needs
Grigoriadis et al., 2019 [145]	Using aMMP8 PoC immunotest for screening prediabetes and diabetes state in periodontal patients	Healthy (*n* = 21) Periodontitis: Stage I/II, grade A-C (*n* = 48)	Mouth rinse samples Capillary blood	aMMP8 assayed by Lateral flow mouth rinse immunoassay test and digital reader ORALyzer^®^ Blood sugar by HbA1c test	PPD and BOP were measured	HbA1c and aMMP8 PoC test can provide dentists with opportunity to diagnose prediabetic and diabetic patients
Räisänen et al., 2019 [146]	Comparing the effectiveness of aMMP8 PoC mouthwash vs BOP in detection pre-/subclinical periodontitis in adolescents	47 adolescents (30 male and 17 female)	Mouth rinse samples	aMMP8 Lateral flow immunoassay test (PerioSafe^®^)	BOP (20% of sites), PPD≥ 4mm, PI, bitewing radiographs for premolars and molars	aMMP8 was twice higher in terms of sensitivity and more accurate than BOP in detecting early stages than BOP
Schmalz et al., 2019 [147]	Investigate the association of aMMP8 with severity of periodontitis, periodontal bacteria, and blood parameters	Periodontitis patients (*n* = 188): Mild (*n* = 50) Moderate (*n* = 111) Severe (*n* = 27)	Mouth rinse samples, blood	aMMP8 measured by Lateral flow immunoassay test (Periomarker^®^)	Based on PPD and CAL, severity of periodontitis was divided into mild, moderate, and severe	aMMP8 was positively associated with severity of periodontitis and anaerobes highly involved in periodontal destruction
Rautava et al., 2020 [123]	Compare the accuracy of aMMP8 PoC immunotest in subjects with and without Crohn’s disease (CD)	Controls (*n* = 47) CD (*n* = 41)	Mouth rinse samples	Lateral flow mouth rinse test (PerioSafe^®^)	Oral mucosal examination for CD-related lesions Caries prevalence by DMFS Gingivitis defined by BOP ≥15% of sites with no CAL or PPD Periodontitis defined by: PPD ≥4 mm, CAL ≥2 mm with or without BOP	Discrimination accuracy of aMMP8 PoC immunotest reduced and diagnosis of periodontitis was compromised with CD patients
Sorsa et al., 2020 [40]	Assessing the usefulness of aMMP8 point of care (PoC) mouthwash in the interpretation of the “Staging” and “Grading” of the new classification system for periodontal disease	Healthy (*n* = 31) Periodontitis (*n* = 119)	Mouth rinse samples	Lateral flow mouth rinse test (PerioSafe^®^) and ORALyzer^®^	Healthy: BOP <10% of sites Periodontitis: Defined according to the new classification system 2018	aMMP8 PoC mouthwash can be integrated for staging and grading of periodontitis
Sorsa et al., 2020 [148]	Investigate the effectiveness of aMMP8 PoC mouthwash in diagnosing peri-implantitis	Healthy subjects (*n* = 20) Peri-implantitis (*n* = 20)	Mouth rinse samples	aMMP8 Lateral flow immunoassay test (ImplantSafe^®^)	Peri-implantitis, diagnosed clinically and radiographically	aMMP8 PoC test correctly diagnosed all healthy and Peri-implantitis cases
Lähteenmäki et al., 2020 [149]	Assessing the accuracy aMMP-8 PISF POC test (ImplantSafe) as compared to other biomarkers of peri-implantitis. Evaluating the value of aMMP-8 lateral-flow PoC technologies in non-invasively monitoring periodontal treatment outcomes	Patients with peri-implantitis (*n* = 26) Healthy control (*n* = 26) Periodontitis patients (*n* = 15)	PISF, mouth rinse samples	aMMP8 Lateral flow immunoassay test (ImplantSafe^®^), (PerioSafe^®^)	Peri-implantitis, diagnosed by presence of PPD ≥4mm, BOP, radiographic bone loss ≥2 mm, PI, FI, mobility index Self-reported oral health (SROH)-questionnaires	aMMP8 PoC test discriminated health from peri-implantitis with higher accuracy than BOP, PMN-elastase, MMP9, TIMP1, and myeloperoxidase SROH can be used as adjunctive diagnostic method but not as alternative for oral fluid biomarkers

MMP, matrix metalloproteinase; IFMA, immunofluorometric assay; ^¶^ PPD, probing pocket depth; CAL, clinical attachment loss; BOP, bleeding on probing; PI, plaque index; BPE, basic periodontal examination; PISF, peri-implant sulcular fluid; CPITN, community periodontal index for treatment need; DMFS, decayed-missed-filled surface index; FI, furcation involvement; TIMP, tissue inhibitor of metalloproteinase.

**Table 3:** Summary of diagnostic biomarker test kits for periodontal diseases [140,152,153].

Assay	Commercial Kit	Sample	Target?
Microbial test kit	PerioScan	Subgingival plaque	Utilizes the BANA test for bacterial trypsin-like proteases
	IAI Pado test	Subgingival plaque	Aa, Pg, Tf, and Td
	Evalusite test	Subgingival plaque, GCF	Aa, Pi, and Pg
	TOPAS	Subgingival plaque, GCF	Toxins derived from anaerobic metabolism and measures GCF protein level
	Omnigene^®^ diagnosctics	Saliva	Pg, Pi, Aa, Fn, Tf, Td, Ec, and Cr
Biochemical test	Prognostik	GCF	Serine proteinases and elastase
	Pocketwatch	GCF	Detects aspartate aminotransferase through colorimetric detection
	Periogard	GCF	Detects the presence of aspartate aminotransferase
	Periocheck	GCF	Detects presence of neutral proteinases (collagenase)
	Progno-Stik	GCF	Elastase
	PerioMarker^®^	GCF	Activated MMP8
	Dip Stick	GCF	MMP8
	Perio 2000	GCF, Subgingival plaque	Sulfides in periodontal pockets
	PerioSafe^®^	GCF, Mouth rinse	Activated MMP8
	ImplantSafe^®^	GCF, PISF	Activated MMP8
	ORALyzer^®^	GCF, Mouth rinse, Saliva	Activated MMP8
	Integrated microfluidic platform for oral diagnostics	Saliva	MMP8
	Oral Fluid NanoSensor test	Saliva	IL-1 and IL-8
	Electronic taste chip	Saliva	C reactive protien
Genetic test kits	GenoType^®^PST^®^	Saliva	Interleukin (IL-1α and IL-1β) genes polymorphism
	MyperioID	Saliva	Genetic variation/polymorphism within the IL-1 gene

GCF: gingival crevicular fluid, PISF: peri-implant sulcular fluid, Aa: Aggregatibacter actinomycetemcomitans, Pg: Porphyromonas gingivalis, Tf: Tannerella forsythia, Td: Treponema denticola, Pi: Prevotella intermedia, Fn: Fusobacterium nucleatum, Ec: Eikenella corrodens, Cr: Campylobacter rectus.
